# Protective Role of Tangshen Formula on the Progression of Renal Damage in *db/db* Mice by TRPC6/Talin1 Pathway in Podocytes

**DOI:** 10.1155/2020/3634974

**Published:** 2020-09-16

**Authors:** Qian Wang, Xuefei Tian, Wei'e Zhou, Yan Wang, Hailing Zhao, Jialin Li, Xuefeng Zhou, Haojun Zhang, Tingting Zhao, Ping Li

**Affiliations:** ^1^Beijing University of Chinese Medicine, Beijing 100029, China; ^2^Beijing Key Laboratory for Immune-Mediated Inflammatory Diseases, Institute of Clinical Medical Sciences, China-Japan Friendship Hospital, Beijing 100029, China; ^3^Section of Nephrology, Department of Internal Medicine, Yale University School of Medicine, New Haven, CT 06510, USA; ^4^Beijing Key Laboratory of Diabetes Research and Care, Center for Endocrine Metabolism and Immune Diseases, Lu He Hospital, Capital Medical University, Beijing 101149, China

## Abstract

Tangshen Formula (TSF) is a Chinese Medicine formula that has been reported to alleviate proteinuria and protect renal function in humans and animals with diabetic kidney disease (DKD). However, little is known about its mechanism in improving proteinuria. The dysregulation of podocyte cell-matrix adhesion has been demonstrated to play an important role in the pathogenesis and progression of proteinuric kidney diseases including DKD. In the present study, the underlying protective mechanism of TSF on podocytes was investigated using the murine model of type 2 DKD *db/db* mice *in vivo* and advanced glycation end products (AGEs)-stimulated primary mice podocytes *in vitro*. Results revealed that TSF treatment could significantly mitigate reduction of podocyte numbers and foot process effacement, reduce proteinuria, and protect renal function in *db/db* mice. There was a significant increase in expression of transient receptor potential canonical channel 6 (TRPC6) and a decrease in expression of talin1 in podocytes of *db/db* mice. The results of AGEs-stimulated primary mice podocytes showed increased cell migration and actin-cytoskeleton rearrangement. Moreover, primary mice podocytes stimulated by AGEs displayed an increase in TRPC6-dependent Ca^2+^ influx, a loss of talin1, and translocation of nuclear factor of activated T cell (NFATC) 2. These dysregulations in mice primary podocytes stimulated by AGEs could be significantly attenuated after TSF treatment. 1-Oleoyl-2-acetyl-*sn*-glycerol (OAG), a TRPC6 agonist, blocked the protective role of TSF on podocyte cell-matrix adherence. In conclusion, TSF could protect podocytes from injury and reduce proteinuria in DKD, which may be mediated by the regulation of the TRPC6/Talin1 pathway in podocytes.

## 1. Introduction

Diabetic kidney disease (DKD) is one of the most common and severe complications of diabetes mellitus and is characterized by proteinuria and renal function impairment. DKD is the major cause of end-stage renal disease (ESRD) worldwide [1]. Accumulating evidence suggests that podocyte injury is a typical manifestation and a core event in the progression of DKD [2]. Podocytes are important components for glomerular filtration barrier integrity and maintenance of size selectivity in protein filtration. Changes in podocyte number and/or structure of foot processes have been demonstrated to be the main cause leading to glomerular proteinuria [3]. Under physiologic conditions, the podocyte actin backbone is well organized to maintain their normal morphology and function. Numerous studies on podocytes have shown that actin-cytoskeletal disorganization and related protein damage induced by multiple pathogenic factors lead to podocyte injury [4, 5]. Cell-matrix adhesion core structure complexes, including integrins/talins/actin, are crucial for maintaining the podocyte actin cytoskeleton. These adhesion complexes allow podocytes to attach tightly to the glomerular basement membrane (GBM) to preserve the integrity of the glomerular filtration barrier. Any deficit in the structural components can disturb podocyte adhesion and lead to the disorganization of the actin cytoskeleton [6].

As a cell-matrix adhesion core structure complex, talin1 impairment can lead to integrin dysfunction, which is associated with a variety of pathologies, including thrombosis, stroke, and cancer metastasis [7]. Tian and colleagues [8] demonstrated that mice lacking talin1 specifically in podocytes displayed severe proteinuria, decreased podocyte adhesion, significant actin-cytoskeletal disorganization, foot process effacement, and progressive renal failure. Transient receptor potential canonical channel 6 (TRPC6) is a nonselective Ca^2+^ channel protein, which has been reported to be closely related to the cleavage of talin1 [9]. Mutation of the *TRPC6* gene in podocytes was first reported by Winn et al. in patients with focal segmental glomerulosclerosis (FSGS), suggesting the potential importance of TRPC6-mediated Ca^2+^ dynamics for podocyte function [10]. More and more studies have shown that aberrant changes in TRPC6 in podocytes lead to proteinuria development and progression of DKD, the mechanism by which may involve the rearrangement of the podocyte actin-cytoskeleton [11, 12]. TRPC6-dependent Ca^2+^ accumulation has been reported to lead to podocyte injury characterizing FSGS through reduced talin1 expression [13]. Furthermore, knockdown of *TRPC6* also results in decreased cleavage of talin1 [9]. However, it is unclear how the podocyte TRPC6/Talin1 signaling pathway affects the development and progression of DKD; thus, this mechanism needs to be investigated.

Tangshen Formula (TSF) is a Chinese Medicine formula that is effective in treating DKD and is based on the empirical evidence of Chinese physicians [14]. TSF is comprised of seven herbs: astragalus root, burning bush twig, rehmannia root, bitter orange fruit, cornus fruit, rhubarb root and rhizome, and notoginseng root and rhizome. Patients with stage III-IV DKD who were treated with one component of TSF, astragalus root injection, experienced improved renal function and decreased proteinuria excretion compared with those in a control group [15]. A decoction made from another TSF component, rehmannia root, was found *in vitro* to dramatically suppress the production of AGEs induced by inflammation [16]. Through liquid chromatography-mass spectrometry (LC-MS), we have successfully detected 40 compounds in TSF and 12 compounds in the serum of TSF-treated mice (supplementary Tables S1 and S2). Six compounds, including loganin, neohesperidin, naringenin, calycosin-7-O-*β*-D-glucoside, naringenine-7-rhamnosidoglucoside, and aloe-emodin, were selected to describe the quality of TSF by comparing the high-performance liquid chromatography (HPLC) fingerprints of 10 sample batches [17, 18]. The results confirmed that these six compounds exhibit renal protective effects [19–26]. Our preliminary multicenter clinical trial [14] and various animal models of DKD [27–29] showed that TSF has significant effects in reducing proteinuria and protecting renal function. However, little is known about its potential mechanism in improving proteinuria and its effects on podocytes. In the present study, we found that TSF mitigated podocyte number loss and foot process effacement reduced proteinuria, and protected renal function in *db/db* mice, a classic genetic model of type 2 diabetes that has clinical and histologic features of DKD resembling changes in humans [30]. Our results further showed that TSF might alleviate proteinuria mediated by the regulation of the TRPC6/Talin1 pathway in podocytes, following enhancement of the podocyte-matrix adhesion ability.

## 2. Materials and Methods

### 2.1. Herbal Formulation and Reagents

TSF (Lot number180408) was prepared and standardized by the Beijing Institute of Clinical Pharmacy, Beijing, China. TSF is composed of seven herbs: astragalus root (*Astragalus membranaceus* (Fisch) Bge.), burning bush twig (*Euonymus alatus* (Thunb.) Siebold.), rehmannia root (*Rehmannia glutinosa* (Gaertn.) Li-bosch.), bitter orange fruit (*Citrus aurantium* L.), cornus fruit (*Cornus officinalis* Sieb & Zucc.), rhubarb root and rhizome (*Rheum palmatum* L.), and notoginseng root and rhizome (*Panax Notoginseng* (Burk.) F. H. Chen) in the ratio of 10 : 5 : 4 : 3.4 : 3 : 2 : 1 (*w*/*w*). TSF was prepared as described in the *Chinese Pharmacopeia*, 2015. Six components, including loganin, neohesperidin, naringenin, calycosin-7-O-*β*-D-glucoside, naringenine-7-rhamnosidoglucoside, and aloe-emodin, were identified in the chemical fingerprint of TSF for quality control as previously described [18]. Irbesartan (J20171089) was purchased from Sanofi Pharmaceutical (Hangzhou, ZJ, China). For the animal experiments, both TSF (0.36 g/mL) and irbesartan (2.25 mg/mL) were dissolved in 0.5% carboxymethylcellulose sodium (CMC-Na).

Antibodies used in this study were as follows: TRPC6 (ab62461) (the specificity of the anti-TRPC6 antibody has been tested by MPC5 cells and *Trpc6* siRNA in Figure S1), WT1 (ab89901), AGE-BSA (ab51995), fibronectin antibody (ab2413), collagen type I (ab34710), and collagen type IV (ab6586), all purchased from Abcam (Cambridge, MA, USA). siRNA against TRPC6 was purchased from Shanghai Generay Biotech (Shanghai, China). Nephrin antibody (GP-N2) was obtained from Progen (Heidelberg, Germany). Talin1 antibody (14168-1-AP), NFATC2 (nuclear factor of activated T cell) antibody (22023-1-AP), NFATC3 antibody (18222-1-AP), integrin *β*1 antibody (26918-1-AP), and GAPDH antibody (60004-1-Ig) were purchased from Proteintech (Wuhan, HB, China). Paxillin antibody (sc-365379) was purchased from Santa Cruz Biotechnology (Dallas, TX, USA). N-2-hydroxyethylpiperazine-N-2-ethane sulfonic acid (HEPES) (15630080), RPMI 1640 medium (no phenol red) (11835030), penicillin-streptomycin (15140122), sodium pyruvate (11360070), sodium bicarbonate (25080094), fetal bovine serum (FBS) (10099141), Alexa Fluor 488 Phalloidin (A12379), Alexa Fluor 594 goat anti-guinea pig secondary antibody (A11076), Alexa Fluor 594 goat anti-rabbit secondary antibody (A11012), and Alexa Fluor 488 goat anti-rabbit secondary antibody (A11008) were purchased from Thermo Fisher Scientific (Waltham, MA, USA). 1-Oleoyl-2-acetyl-sn-glycerol (OAG) (O6754), Hanks' balanced salt solution (HBSS) (H6648), and collagen type I solution from rat tail (C3867) were purchased from Sigma-Aldrich (St. Louis, MO, USA). Collagenase A (11088793001) and DNase (04716728001) were purchased from Roche (Mannheim, Germany). CMC-Na (C8621) was purchased from Solarbio (Beijing, China). ELISA Quantitation Set kit (E101) was obtained from Bethyl Laboratories (Montgomery, TX, USA). Fluo-4-AM (S1060), ionomycin (S1672), EDTA (ST066), and MTT Cell Proliferation and Cytotoxicity Assay Kit (C0009) were purchased from Beyotime Biotechnology (Shanghai, China). SAR7334 (HY-15699) was purchased from MedChemExpress (Shanghai, China).

### 2.2. Assay of the Compounds in TSF and in the Mouse Serum Treated with TSF Determined by UHPLC-MS/MS

The 1 g of dried TSF compound prescription power was extracted with methanol-water (25 mL, 75 : 25, *v*/*v*) using an FS30 ultrasonic sonicator (Fisher Scientific, Pittsburg, PA, USA) at 40 kHz and 100 W for 30 minutes at room temperature. A 10 *μ*g of the extract was injected onto the analytical column for analysis. For the preparation of mouse serum sample treated with TSF, the 8-week-old male C57BL/6J mouse was gavaged with TSF at the dosage of 2.4 g/kg body weight, the serum sample was collected after 24 hours administration. The 150 *μ*L of mouse serum sample was mixed with 400 *μ*L methanol, following by centrifuging at 14480 g for 10 minutes at 4°C. The supernatant was evaporated to dryness by Centrivap Concentrator (LABCONCO, USA), then 400 *μ*L methanol was added. After centrifuging at 14480 g for 20 minutes at 4°C, supernatant was collected for analysis.

The prepared samples were analyzed using the ultrahigh performance liquid chromatography-tandem mass spectrometry (UHPLC-MS/MS). Briefly, UHPLC-MS/MS analysis was performed using Q Exactive Plus High Resolution Mass Spectrometer (THERMO, USA) equipped with Ultimate 3000 (DIONEX, THERMO, USA), and Acquity™ UPLC BEH C18 column (2.1 × 100 mm, 1.7 *μ*m) (Waters, USA). The optimized chromatographic conditions were achieved at a flow rate of 0.3 mL/min with a mobile phase consisting of acetonitrile (mobile phase A) and 0.1% formic acid solution (mobile phase B). The diode array detector (DAD) was set at 280, 254 nm for real-time monitoring of the peak intensity and full spectra (190-650 nm). The following conditions were applied for MS: capillary temperature 320°C, spray voltage 3.5 kV for ESI+analysis; and capillary temperature 320°C, spray voltage -3.5 kV for ESI-analysis. The mass scan range was set to 100-1500. The resolution of the Orbitrap was set to 70000. The resolution of dd-MS2 was set to 17500; CE was set to 20 : 40 : 60. The peak picking and alignment were processed using Sieve software (V1.2, Thermo Fisher Scientific, USA) applying a mass width of 0.02 Da and a retention time width of 0.5 minutes.

### 2.3. Animals and Experimental Design

Eight-week-old male C57BLKS/J^Lepr^*db/db* mice (*n* = 18) weighing 42.68 ± 0.66 g and *db/m* mice (*n* = 6) weighing 23.97 ± 0.49 g were purchased from the Peking University Laboratory Animal Center (Beijing, China). Since the misty (*m*) gene has a greater effect on the metabolism of mice, *m/m* mice are 8% shorter and weigh 15% less than controls and have less inguinal adipose mass and complete loss of brown fat [31]. Therefore, the *db/m* mice are usually used as the control group in *db/db* mice studies [32]. All mice were maintained inhouse under the following specific pathogen-free conditions: 22 ± 2°C controlled temperature, 65-75% relative humidity, a regular 12-hour light/dark cycle, and received standard laboratory chow and water *ad libitum*. After 2 weeks of acclimation, the *db/m* mice were used as controls (designated *db/m*). *db/db* mice were randomly assigned into three groups (*n* = 6, per group): *db/db* mice (designated *db/db*), *db/db* mice treated with TSF (3.6 g/kg/day) (designated *db/db* + TSF), and *db/db* mice treated with irbesartan (22.5 mg/kg/day) (designated *db/db* + Irbesartan). The *db/m* and *db/db* groups were each given equal volume of 0.5% CMC-Na, gavage administration. All drugs were administered once daily by gastric gavage. Dose of TSF was adjusted based on our previous studies and standard conversion formulas [14, 33]. Body weight was recorded weekly during the study period. Urine was collected at the beginning of treatment and the 12th week. The mice were sacrificed after 12 weeks of treatment. After overnight fasting, the serum and tissue samples were collected rapidly for further analysis. All experimental animal procedures were in accord with the National Institutes of Health *Guide for the Care and Use of Laboratory Animals* (2011 edition). The protocol was approved by the Ethics Committee of the China-Japan Friendship Institute of Clinical Medical Sciences (Approval no.13005).

### 2.4. Biochemistry Measurements

Mouse urine samples were collected using metabolic cages (Fengshi Inc., Suzhou, JS, China) [27]. Urine albumin was quantified in duplicates using the Mouse Albumin ELISA Kit according to the manufacturer's protocol (Bethyl Laboratories). Urine creatinine was measured in duplicate for each sample using an automatic analyzer (Abbott Diagnostics, Abbott Park, IL, USA).

### 2.5. Histologic Assessment of Glomerular Injury

Kidney tissues were fixed in 10% formalin, embedded in paraffin sections (2-3 *μ*m), and stained with periodic acid-Schiff (PAS) and periodic acid-silver methenamine (PASM). Kidney ultrastructure was detected by transmission electron microscopy (TEM) (JEOL-100CXII, JEOL, Tokyo, Japan). Kidney tissues (1 mm^3^) were fixed with 2.5% glutaraldehyde at 4°C for 24 hours and embedded in epoxy resin. Ultrathin sections were sliced and stained with uranyl acetate and lead citrate. Twenty glomerular cross-sections (with the vascular pole and the urinary pole on the same plane) per sample were randomly selected under a 400x light microscope (Olympus, Tokyo, Japan), and the areas of each glomerulus and mesangial matrix were measured using Image-Pro Plus 6.0 software (Media Cybernetics, Warrendale, PA, USA) by two pathologists in a double-blinded manner. Thirty glomerular capillaries per sample were randomly selected (magnification 12000x) to measure GBM thickness, podocyte foot process width, and the number of foot processes per micron in a double-blinded manner [34, 35].

### 2.6. Immunohistochemistry and Immunofluorescence

Paraffin-embedded mice kidney sections were deparaffinized with xylene following hydration with ethanol. Antigen retrieval was induced by microwave following washing with 1× phosphate buffer saline (PBS) as previously described [8]. Sections were incubated overnight at 4°C with anti-fibronectin antibody (1 : 500), anti-collagen type I antibody (1 : 1000), anti-collagen type IV antibody (1 : 400), anti-nephrin antibody (1 : 2000), and anti-WT1 antibody (1 : 1000). Subsequently, the sections were incubated with secondary antibodies and counterstained with hematoxylin for the nuclei. Images were captured under the light microscope and then measured by Image-Pro Plus 6.0 software. For immunofluorescence, paraffin-embedded kidney sections were incubated in blocking buffer (5% BSA in 1× PBS) for 1 hour at room temperature and stained with antibodies in the following concentration: anti-nephrin antibody (1 : 100) and secondary antibody Alexa Fluor 594 goat anti-guinea (1 : 200). Costaining was with the following antibodies: anti-TRPC6 antibody (1 : 100), anti-talin1 antibody (1 : 100), anti-WT1 antibody (1 : 100), and secondary antibody Alexa Fluor 488 goat anti-rabbit (1 : 200). For cell immunofluorescence, primary mice podocytes on collagen type I-coated coverslips were washed with 1× PBS and fixed with 4% paraformaldehyde for 15 minutes. The cells were permeated with 0.5% Triton X-100 for 20 minutes at room temperature. The cells were blocked with 5% BSA in 1× PBS for 1 hour and stained with antibodies in the following concentration: anti-TRPC6 antibody (1 : 100), anti-WT1 antibody (1 : 100), anti-Talin1 antibody (1 : 50), anti-paxillin antibody (1 : 100), anti-NFATC2 antibody (1 : 100), anti-NFATC3 antibody (1 : 100), and secondary antibody Alexa Fluor 488 goat anti-rabbit (1 : 300). The resulting images were obtained by a fluorescent inverted microscope (Olympus).

### 2.7. Isolation of Primary Mice Podocytes

The protocol for primary mice podocytes culture isolated from newborn mice was performed as previously described [8]. The freshly removed kidneys from the C57BL/6J newborn mice (Beijing HFK Bioscience, Beijing, China) were minced into very small pieces in a 10 cm dish. After digestion for 30-45 minutes in a collagenase solution (5 mg/mL collagenase A and 1 : 10 DNase) at 37°C and 5% CO_2_ incubation, the resulting suspension was filtered with a 70 *μ*m BD Falcon cell strainer (Corning Life Sciences, Corning, NY, USA). The filtered solution (glomerular cells) was then plated onto the 10 cm culture dish coated with collagen type I to total 1640 RPMI medium with 10% FBS, 1% penicillin-streptomycin, 10 mM HEPES, 0.075% sodium bicarbonate, and 1 mM sodium pyruvate. The primary mice podocytes were harvested by separating the glomerular cells with 0.25% trypsin (Invitrogen) and then sieving through a 40 *μ*m BD Falcon cell strainer. Cells were cultured in a humidified incubator with 5% CO_2_ at 37°C and used at passages 1 or 2 for all experiments. The purity of the primary mice podocytes was 95% as determined by immunofluorescence staining with the WT-1 antibody, a specific marker of podocytes [36].

### 2.8. Cell Treatments

To investigate the role of AGEs, the primary mice podocytes were exposed to AGE-BSA for 24 hours sequentially at 60, 80, 100, and 120 *μ*g/mL. Equal amounts of BSA were added to the control cells. After successfully producing the podocyte injury model induced by AGEs, the cells were treated with a series of concentrations (250, 500, and 750 *μ*g/mL) of TSF for 24 hours with AGE-BSA (100 *μ*g/mL). The cells cultured with BSA (100 *μ*g/mL) served as controls. SAR7334 (0.1 *μ*mol/L) was used as a TRPC6 inhibitor while OAG (100 *μ*mol/L) as a TRPC6 agonist.

### 2.9. Determination of Cell Viability

The effect of TSF on cell viability was determined using the 3-(4,5-dimethylthiazol-2-yl)-2,5-diphenyltetrazolium bromide (MTT) assay. After being starved with 1640 RPMI medium without FBS overnight, the primary mice podocytes (2 × 10^3^ cells/well) growing in 96-well plates were incubated with BSA (100 *μ*g/mL) and TSF (0, 100, 250, 500, 750, and 1000 *μ*g/mL) for 24 hours. Each well was incubated with 10 *μ*L MTT solution (5 mg/mL) for 4 hours in a cell culture incubator. Subsequently, 100 *μ*L formazan lysate was added to each well and measured at 570 nm with a microplate reader (Thermo Fisher Scientific). Cell viability was calculated as a percentage according to the manufacturer's instructions.

### 2.10. Wound Healing Assay

The primary mice podocytes (1 × 10^6^ cells/well) were incubated in 6-well plates and were starved for 24 hours. Once the cells reached 100% confluence, an artificial linear wound (scratch) was introduced using a sterile 200 *μ*L pipette tip. Cells were allowed to heal for 24 hours. The percentage of wound closure was calculated as [(Area T_24 hours_ − Area T_0_)/Area T_0_] using the ImageJ software 1.8.0 (National Institutes of Health, Bethesda, MD, USA) [37].

### 2.11. Quantitative Analysis of Changes in the Actin Cytoarchitecture of Primary Mice Podocytes

Changes in actin cytoarchitecture of primary mice podocytes were analyzed as previously described [8]. The multiplicity of different phalloidin staining patterns was grouped into four major classes and used for scoring as follows: Type A: more than 90% of cell area filled with thick cables; Type B: at least two thick cables running under the nucleus and the rest of the cell filled with fine cables; Type C: no thick cables, but some fine cables present; Type D: no cables visible in the central area of the cell. All slides were scored in a double-blinded manner; thirty cells in each slide were analyzed in three separate experiments.

### 2.12. Intracellular Ca^2+^ Determination

Intracellular Ca^2+^ concentration was determined using the Ca^2+^ fluorescent probe Fluo-4-AM. Primary mice podocytes in each group were washed three times with HBSS (no calcium, no magnesium, no phenol red) and incubated with Fluo-4-AM (5 *μ*mol/L in RPMI 1640 medium) at 37°C for 45 minutes. Podocytes were trypsinized with 0.25% trypsin-EDTA, followed by centrifugation at 200 × g for 5 minutes. Intracellular Ca^2+^ level was measured and calculated using the FACScan flow cytometer (BD Biosciences, San Jose, CA, USA) [38]. Then, the cells were incubated with 5 *μ*mol/L ionomycin in a zero Ca^2+^ bath (0 mmol/L Ca^2+^, 5 mmol/L EDTA) and 5 *μ*mol/L ionomycin in saturating Ca^2+^ solution (5 mmol/L CaCl_2_), to measure minimal and maximal responses to Ca^2+^ [39].

### 2.13. Western Blot Analysis

Protein samples of kidney tissue and cells were extracted in RIPA lysis buffer (EpiZyme, Shanghai, China) at 4°C. Concentrations of protein were determined using BCA assay. Equal amounts of total protein were denatured for 10 minutes at 95°C. The protein lysates (25 *μ*g) were separated in 10% or 12% polyacrylamide gels (EpiZyme) and transferred onto 0.45 *μ*m polyvinylidene fluoride (PVDF) membranes (Millipore Sigma, Darmstadt, Germany). After blocking with 5% BSA in 1× PBS, membranes were incubated with antibodies overnight at 4°C in the following concentrations: anti-nephrin antibody (1 : 500), anti-WT1 antibody (1 : 500), and anti-talin1 antibody (1 : 500). Blots were detected using enhanced chemiluminescence (Amersham Pharmacia Biotech, Buckinghamshire, UK) with the ChemiDoc XRS system (Bio-Rad, Hercules, CA, USA). Finally, the protein bands were quantified using the ImageJ software.

### 2.14. Statistical Analysis

All data were presented as the mean ± SEM. Numbers for each experiment are displayed in the Methods or the figure legends. Statistical difference between two groups was evaluated by a 2-tailed *t*-test. Multiple-group comparisons were evaluated using one-way ANOVA followed by Dunnett's multiple-comparisons tests. Statistical analysis was performed by the GraphPad Prism 6.0 software (San Diego, CA, USA). A *P* value less than 0.05 was considered statistically significant.

## 3. Results

### 3.1. TSF Reduced Proteinuria and Attenuated Renal Injury in *db/db* Mice

**Figure 1:**
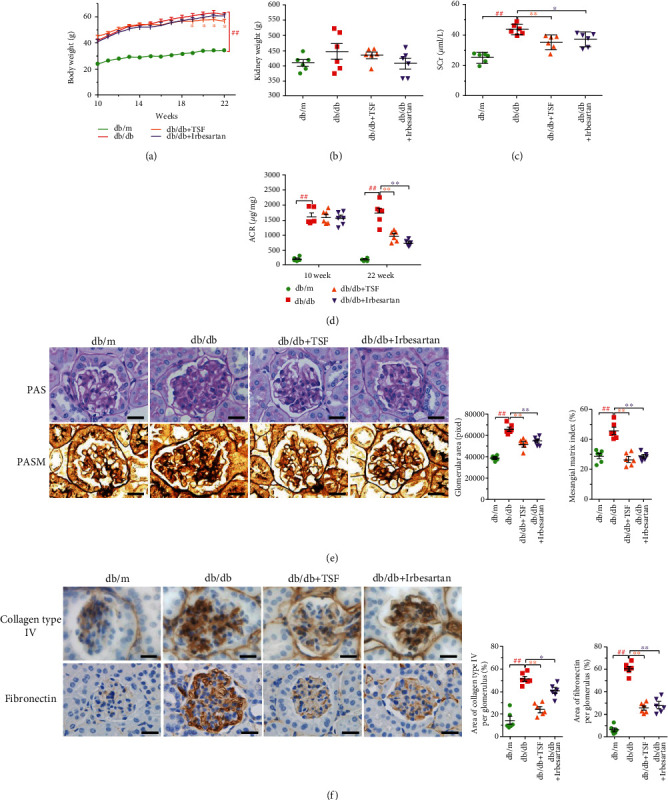
TSF reduced proteinuria and alleviated renal injury in *db/db* mice. (a) Body weight was recorded each week (*n* = 6). (b) Kidney weight of mice in each group at 22 weeks of age (*n* = 6). (c) Urinary albumin to creatinine ratio (ACR) (*n* = 6). (d) Serum creatinine in each group (*n* = 6). (e) Representative PAS staining (scale bars, 20 *μ*m) and quantitative analysis of glomerular hypertrophy and mesangial expansion (*n* = 6); representative PASM staining (scale bars, 20 *μ*m). (f) Representative immunohistochemical staining and quantitative analysis of collagen type IV and fibronectin (scale bars, 20 *μ*m) (20 glomeruli were randomly evaluated per section) (*n* = 6). The data were expressed as the mean ± SEM. ^#^*P* < 0.05, ^##^*P* < 0.01 vs. *db/m* group; ^∗^*P* < 0.05, ^∗∗^*P* < 0.01 vs. *db/db* group. Statistically analyzed via a one-way ANOVA with Dunnett's correction.

During the 12-week intervention with TSF or irbesartan, there were no obvious side effects observed in the *db/db* mice. The body weights between the mice of *db/db* group and the mice of *db/db* + TSF group were similar at the age of 10 weeks. TSF treatment significantly reduced the body weights of *db/db* mice compared with the *db/db* mice without treatment starting from age of 19 weeks till the end of the study. However, there were no significant changes in the body weights of *db/db* mice treated with irbesartan observed (Figure 1(a)). There were no changes in the kidney weights either TSF-treatment or irbesartan-treatment in the *db/db* mice compared to vehicle-treatment control *db/db* mice (Figure 1(b)).

Renal damage in *db/db* mice was assessed by the urine albumin-to-creatinine ratio (ACR) assay and serum creatinine levels. Compared with mice of 10 weeks of age, ACR in *db/db* mice was significantly decreased after 12 weeks of TSF treatment (Figure 1(c)). Moreover, TSF significantly reduced serum creatinine levels in *db/db* mice (Figure 1(d)). Irbesartan-treated *db/db* mice also showed significant improvement in ACR and serum creatinine. The typical pathologic damage changes in *db/db* mice, including glomerular hypertrophy, mesangial expansion, thickened GBM, and abnormal deposition of fibronectin, collagen type IV, and collagen type I, were relieved by TSF or irbesartan treatment (Figures 1(e), 1(f), and S2). Therefore, TSF could effectively alleviate proteinuria and renal injury in *db/db* mice, which is consistent with our previously reported results [29, 40].

### 3.2. TSF Improved Podocyte Injury, Reduced Expression of TRPC6 and Enhanced Expression of talin1 in *db/db* Mice Podocytes

**Figure 2:**
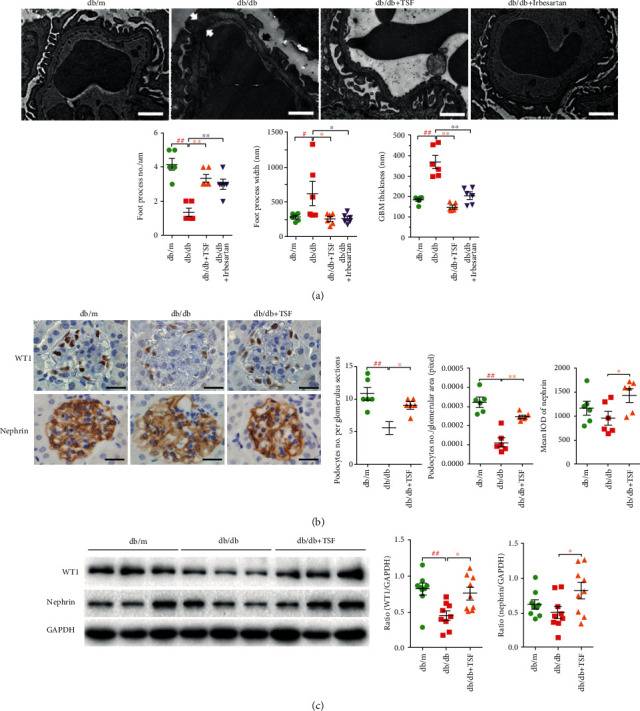
TSF reduced foot process effacement, prevented WT1 from loss in *db/db* mice. (a) Representative transmission electron micrographs (scale bars, 1 *μ*m) illustrate foot process effacement (black arrow) and GBM thickness (white arrow); the number and width of foot process and GBM thickness were subsequently calculated (*n* = 6). (b) Representative immunohistochemical staining and quantitative analysis of WT1 and nephrin (scale bars, 20 *μ*m) (20 glomeruli were randomly evaluated per section, *n* = 6). (c) Immunoblotting for WT1 and nephrin; quantification of the gels is shown on the right (*n* = 3 experiments). The data were expressed as the mean ± SEM. ^#^*P* < 0.05, ^##^*P* < 0.01 vs. *db/m* group; ^∗^*P* < 0.05, ^∗∗^*P* < 0.01 vs. *db/db* group. Statistically analyzed via a one-way ANOVA with Dunnett's correction.

To investigate the ultrastructural changes in the podocytes, transmission electron microscopy was performed. The results showed that there were a severe foot process effacement and an increased GBM thickness in the *db/db* mice, which was strikingly improved by either TSF or irbesartan treatment at a similar effect (Figure 2(a)). Consistent with the PASM results (Figure 1(e)), the thickened GBM in *db/db* mice under the electron microscopy also showed significant relief after TSF treatment. We further studied the expression of WT1 and nephrin, two specific podocyte markers, on kidney tissue of *db/db* mice. Our results showed that there was a significant decrease in WT1 expression, and no significant change in nephrin either in protein expression or localization (Figures 2(b) and 2(c)). Given the presenting characteristics of early DKD in *db/db* mice, it was speculated that partial foot process effacement of *db/db* mice had not seriously affected the structure of the slit diaphragm (SD) of podocytes and the related protein nephrin.

**Figure 3:**
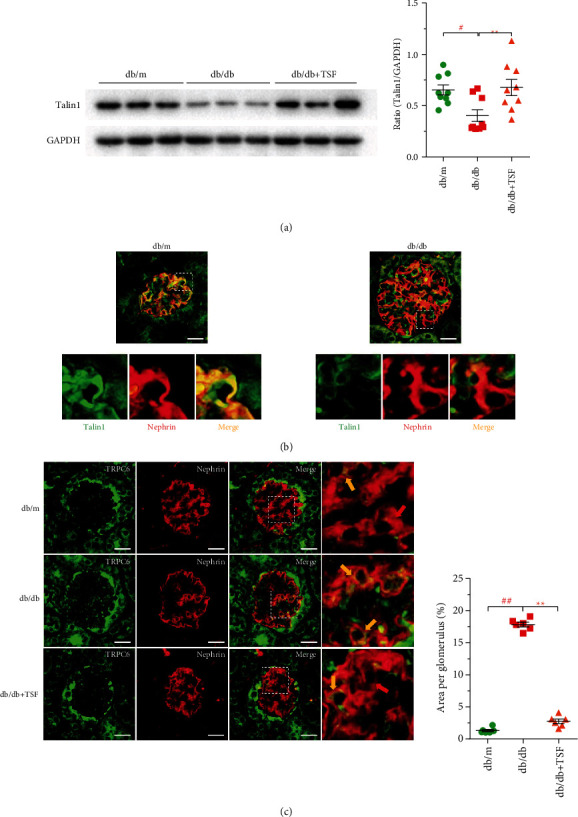
TSF protects podocyte-associated protein talin1 in *db/db* mice, which may involve increased expression of TRPC6 in podocytes. (a) Immunoblotting and quantitative analysis of talin1 (*n* = 3 experiments). (b) Immunofluorescence costaining (scale bars, 25 *μ*m) of nephrin (red) and talin1 (green) showed that talin1 had a specific deletion in podocytes. (c) Immunofluorescence costaining (scale bars, 25 *μ*m) of nephrin (red; red arrow) and TRPC6 (green) showed that increased expression of podocyte TRPC6 in *db/db* mice (yellow arrow) compared to *db/m* mice and *db/db* + TSF mice (*n* = 6). Fluorescent images were collected and assessed using a high-content screening system. The data were expressed as the mean ± SEM. ^#^*P* < 0.05, ^##^*P* < 0.01 vs. *db/m* group; ^∗^*P* < 0.05, ^∗∗^*P* < 0.01 vs. *db/db* group. Statistically analyzed via a one-way ANOVA with Dunnett's correction.

Considering reduction of WT1 represented decrease in podocyte numbers, we examined the podocyte-associated protein talin1, which is a crucial part of the podocyte-matrix adhesion structure. Results showed that there was a significant loss in talin1 protein in *db/db* mice and that TSF could prevent its loss (Figure 3(a)). Since talin1 expresses ubiquitously, we observed the change in talin1 in podocytes by costaining with nephrin. We found that talin1 loss was mainly concentrated in podocytes in *db/db* mice (Figure 3(b)). Verheijden et al. [13] reported that loss of talin1 is closely related to the abnormal expression of TRPC6. Based on this, we attempted to determine the potential changes in the expression of TRPC6 on podocytes by costaining with nephrin. Our results showed there was increased TRPC6 expression in *db/db* mice podocytes, which could be mitigated by TSF treatment (Figure 3(c)). This suggested that podocyte injury in *db/db* mice could be attributed to the loss of podocyte-specific talin1 protein, which may be associated with the increase in TRPC6 expression. Thus, TSF had a significant protective effect on podocyte talin1 in *db/db* mice.

### 3.3. TSF Alleviated Cell Migration and Actin-Cytoskeletal Disorganization in AGEs-Stimulated Primary Mice Podocytes

**Figure 4:**
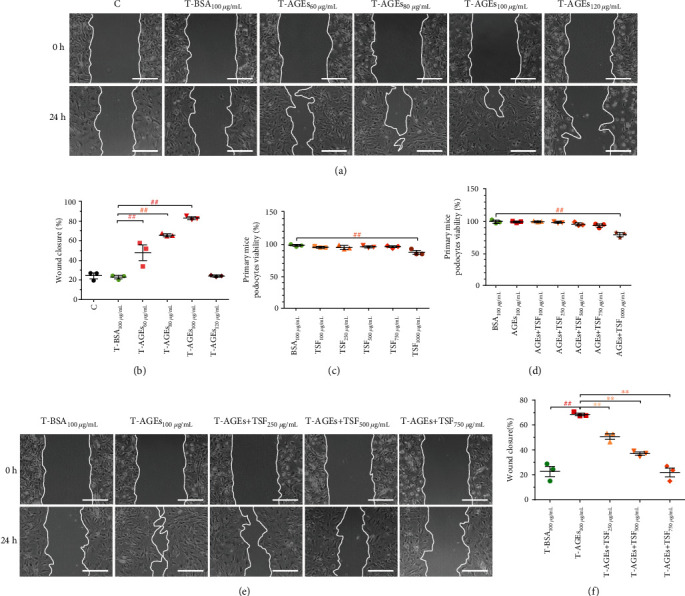
TSF attenuated the increased cell migration in AGEs-stimulated primary mice podocytes. (a, b) Wound healing assay and quantitative analysis using different doses of AGEs for 24 hours to observe the effect on primary mice podocytes (scale bars, 100 *μ*m) (*n* = 3 experiments). (c, d) Viability of primary mice podocytes was determined with MTT assay (*n* = 3 experiments). (e, f) Wound healing assay and quantitative analysis using different doses of TSF for 24 hours (scale bars, 100 *μ*m) (*n* = 3 experiments). The data were expressed as the mean ± SEM of three independent experiments performed in triplicate. ^#^*P* < 0.05, ^##^*P* < 0.01 vs. BSA group; ^∗^*P* < 0.05, ^∗∗^*P* < 0.01 vs. AGEs group. Statistically analyzed via a one-way ANOVA with Dunnett's correction.

To further understand the role of talin1 and TRPC6 in podocytes and the mechanism of TSF, we continued our investigation by culturing primary mice podocytes *in vitro*. AGEs are involved in the pathogenesis of DKD and are closely related to the extent of podocyte injury [41, 42]. In combination with the AGEs deposition found in the kidney tissue of *db/db* mice, we used AGEs to establish a primary mice podocyte injury model *in vitro*. Loss of podocyte talin1 results in a reduction in cell adhesion, an increase in cell migration, and actin-cytoskeletal disorganization [8]. TRPC6-dependent Ca^2+^ accumulation has been found to cause podocyte injury through the loss of talin1 in human and experimental FSGS [13]. Therefore, we hypothesize that the lack of talin1 in type 2 DKD model *db/db* mice may also occur following TRPC6 activation, and TSF may have a certain degree of therapeutic effect through this mechanism. Cells lacking talin1 have impaired abilities on reduced cell adhesion, spreading, and migration [43]. Moreover, the changes in different phalloidin staining patterns in podocytes have been shown to be associated with podocyte migration ability [44]. We observed their migration ability through the wound healing assay after starving for 24 hours. Given that podocytes are terminally differentiated epithelial cells lacking the proliferative ability and our results, the effects of podocyte proliferation on the wound healing assay are less likely especially under the same experimental conditions. The most obvious migration occurred in primary mice podocytes after AGEs stimulation at a dose of 100 *μ*g/mL for 24 hours (Figures 4(a) and 4(b)). The cytotoxicity of TSF on primary mice podocytes was analyzed using MTT at a concentration range of 100 *μ*g/mL to 1000 *μ*g/mL. Results showed that TSF treatment at a concentration of up to 750 *μ*g/mL had no effect on the cells (Figure 4(c)). MTT results revealed that TSF treatment with AGEs (100 *μ*g/mL) at a concentration of up to 750 *μ*g/mL had no effect on primary mice podocytes (Figure 4(d)). TSF mitigated podocyte migration induced by AGEs in a dose-dependent manner (250, 500, and 750 *μ*g/mL) (Figures 4(e) and 4(f)).

**Figure 5:**
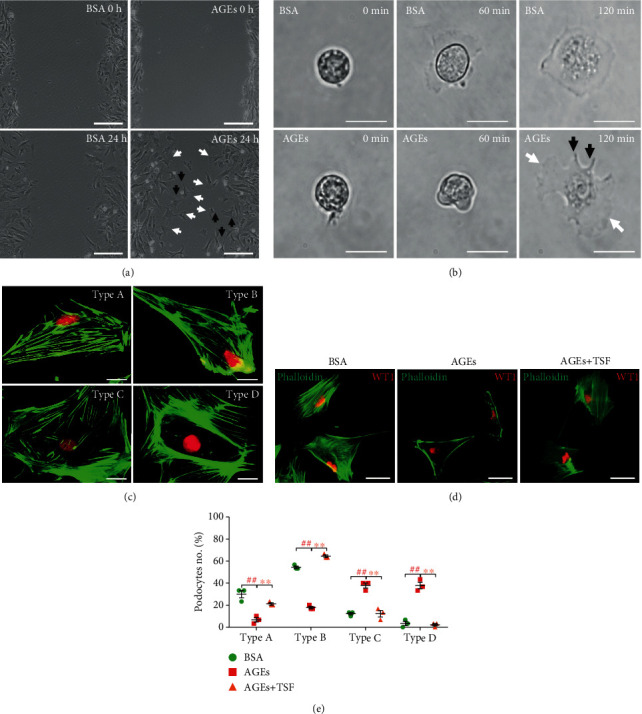
TSF inhibited actin-cytoskeletal disorganization in AGEs-stimulated primary mice podocytes. (a) Morphology of primary mice podocytes in wound healing assay (scale bars, 100 *μ*m). (b) Time-lapse images of primary mice podocytes (scale bars, 50 *μ*m). Lamellipodia are marked with white arrows and filopodia with black arrows. (c) Representative images of different types of phalloidin staining patterns observed in isolated control podocytes (scale bars, 25 *μ*m). (d) Representative immunofluorescence stained with phalloidin and WT1 showed actin-cytoskeletal disorganization in AGEs-stimulated primary mice podocytes which could be relieved by TSF (scale bars, 50 *μ*m). (e) Quantitative analysis of the percentage of four types of phalloidin staining patterns in (d) by a blinded observer (30 cells evaluated per experiment, *n* = 3 experiments). The data were expressed as the mean ± SEM of three independent experiments performed in triplicate. ^#^*P* < 0.05, ^##^*P* < 0.01 vs. BSA group; ^∗^*P* < 0.05, ^∗∗^*P* < 0.01 vs. AGEs group. Statistically analyzed via a one-way ANOVA with Dunnett's correction.

Based on this, we observed podocyte morphology as related to migration. Compared with the control group, podocytes in the AGEs-stimulated group appeared to have the more obvious formation of lamellipodia and filopodia (Figures 5(a) and 5(b)), which are associated with increased cell motility and actin rearrangement.

Immunofluorescence labeling of the podocyte nucleus with WT1 and F-actin with phalloidin showed that AGEs caused actin-cytoskeletal disorganization in cultured primary mice podocytes. The AGEs-stimulated primary mice podocytes had fewer cells with types A (>90% of cell area filled with thick cables) and B (at least two thick cables running under the nucleus and rest of cell area filled with fine cables) staining patterns and more cells in type C (no thick cables, but some cables present), and type D (no cables visible in the central area of the cell) pattern when compared with control podocytes, and these effects were decreased by TSF (500 *μ*g/mL) (Figures 5(c) and 5(d)). In short, TSF inhibited cell migration and attenuated actin-cytoskeletal disorganization in primary mice podocytes stimulated by AGEs.

### 3.4. TSF Alleviated Podocyte Injury by Improving Cell-Matrix Adhesion Ability through Regulation of TRPC6/Talin1 Pathway

**Figure 6:**
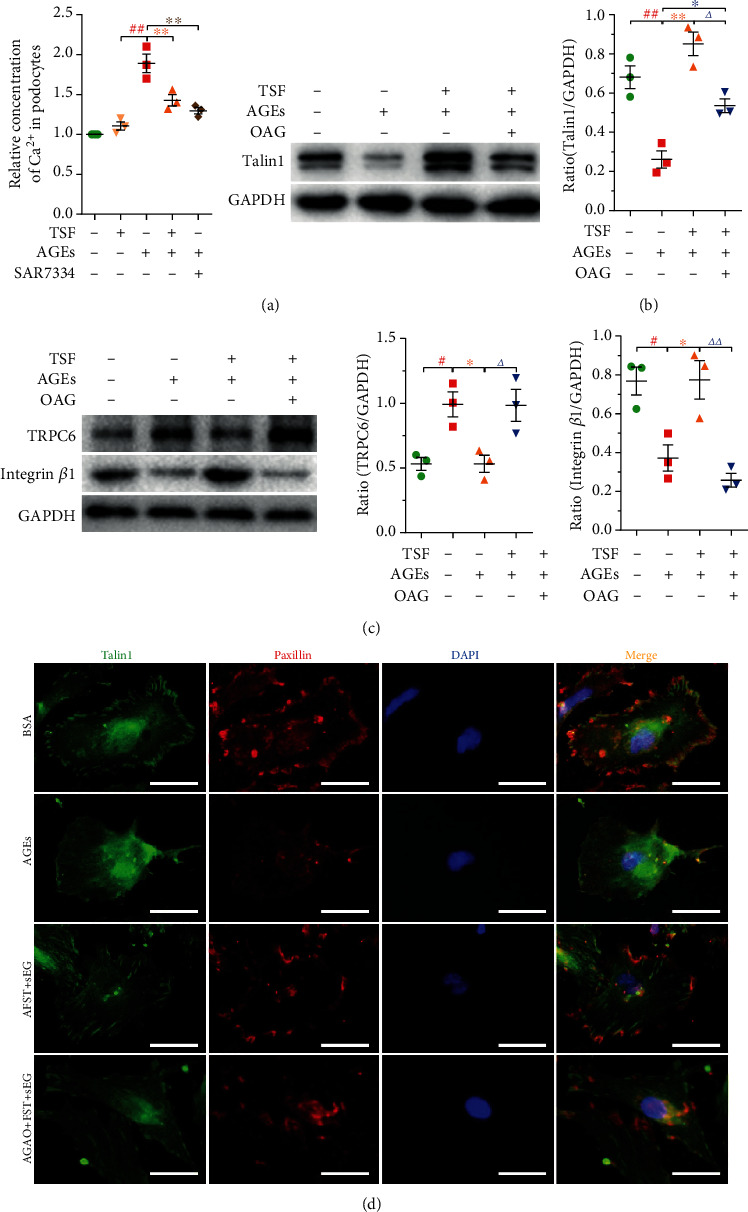
TSF improved TRPC6-dependent Ca^2+^ accumulation-mediated reduction of focal adhesions in AGEs-stimulated primary mice podocytes. (a) Effects of TSF or SAR7334 on intracellular Ca^2+^ level of AGEs-induced primary mice podocyte injury (*n* = 3 experiments). (b) Immunoblotting and quantitative analysis of talin1 (*n* = 3 experiments). (c) Immunoblotting and quantitative analysis of TRPC6 and integrin *β*1 (*n* = 3 experiments). (d) Representative immunofluorescence stained with talin1 (green) and paxillin (red) (scale bars, 50 *μ*m). The data were expressed as the mean ± SEM of three independent experiments performed in triplicate. ^#^*P* < 0.05, ^##^*P* < 0.01 vs. BSA group; ^∗^*P* < 0.05, ^∗∗^*P* < 0.01 vs. AGEs group; *^Δ^P* < 0.05, *^ΔΔ^P* < 0.01 vs. AGEs + TSF group. Statistically analyzed via a one-way ANOVA with Dunnett's correction.

**Figure 7:**
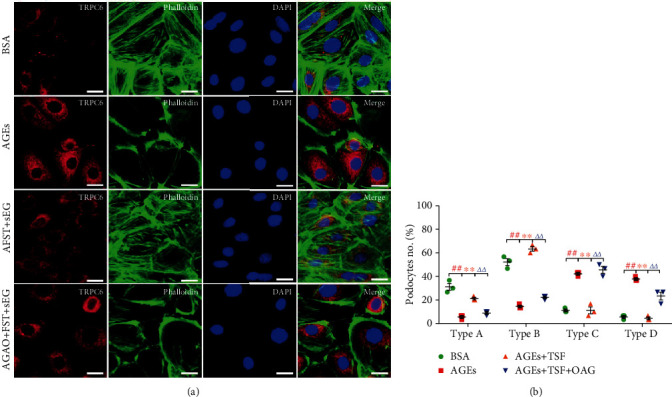
TSF alleviated TRPC6-dependent Ca^2+^ accumulation-mediated actin-cytoskeletal disorganization in AGEs-stimulated primary mice podocytes. (a) Representative immunofluorescence stained with phalloidin and TRPC6 (scale bars, 25 *μ*m). (b) Quantitative analysis of the percentage of four types of phalloidin staining patterns in (a) by an observer blinded to the group (30 cells evaluated per experiment, *n* = 3 experiments). The data were expressed as the mean ± SEM of three independent experiments performed in triplicate. ^#^*P* < 0.05, ^##^*P* < 0.01 vs. BSA group; ^∗^*P* < 0.05, ^∗∗^*P* < 0.01 vs. AGEs group; *^Δ^P* < 0.05, *^ΔΔ^P*<0.01 vs. AGEs + TSF group. Statistically analyzed via a one-way ANOVA with Dunnett's correction.

We explored the relationship between AGEs and TRPC6 by calculating the relative concentration of Ca^2+^ in AGEs-stimulated primary mice podocytes using Ca^2+^-sensitive dye fluo-4-AM. Results showed that compared with the BSA group, the BSA+TSF group had no effect on podocyte intracellular Ca^2+^ concentration. However, there was a significant increase in Ca^2+^ level in AGEs-treated podocytes compared with the control group, while in the groups that were pretreated with TSF and SAR7334 (0.1 *μ*mol/L), a potent and specific cell-membrane permeable inhibitor of TRPC6 [45], there was a significant decrease in Ca^2+^ level compared with AGEs-treated podocytes (Figure 6(a)). Also, there was a decrease in talin1 in AGEs-stimulated primary mice podocytes, accompanied by an increase in the expression of TRPC6 (Figures 6(b) and 6(c)). The loss of talin1 has been shown to affect the expression of some focal adhesions; thus, we initially evaluated the expression of integrin *β*1 and paxillin, two kinds of intracellular anchor proteins in cell-matrix adhesion that can bind directly to actin. The results showed a decrease in integrin *β*1 and paxillin in AGEs-treated podocytes (Figures 6(c) and 6(d)). TSF protected talin1, integrin *β*1, and paxillin from loss and reduced expression of TRPC6, and all phenomena could be attenuated by OAG (100 *μ*mol/L), agonist of TRPC6. These results showed that TSF could improve cell-matrix adhesion ability and reduce TRPC6-dependent Ca^2+^ accumulation in AGEs-stimulated primary mice podocytes. We also observed that the protective effect of TSF on podocyte actin fibers was inhibited by OAG (Figures 7(a) and 7(b)). This indicates that TRPC6 plays an important role in maintaining the stability of the podocyte actin-cytoskeleton and that TSF could alleviate TRPC6-dependent Ca^2+^ accumulation mediated actin-cytoskeletal disorganization and increase the cell-matrix adhesion ability in podocytes.

**Figure 8:**
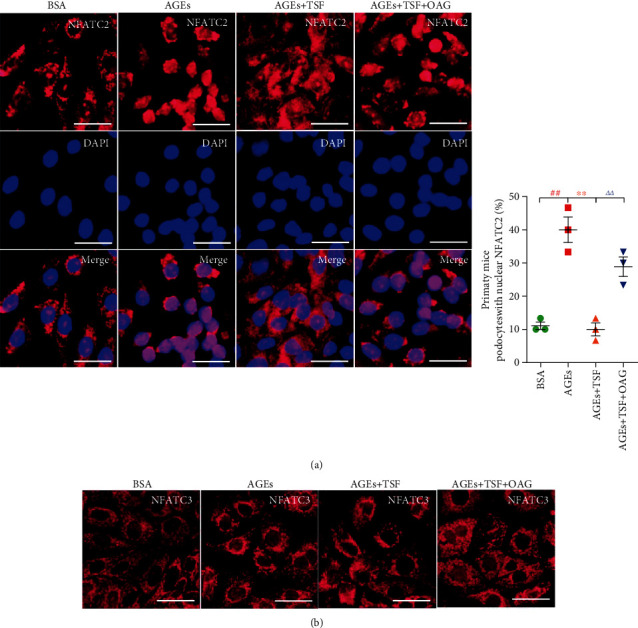
TSF alleviated NFATC2 nuclear translocation in AGEs-stimulated primary mice podocytes. (a) Representative immunofluorescence staining and quantitative analysis of NFATC2 (scale bars, 50 *μ*m; 30 cells evaluated per experiment, *n* = 3 experiments). Nuclei were stained with DAPI. (b) Immunofluorescence staining for NFATC3 (scale bars, 50 *μ*m). The data were expressed as the mean ± SEM. ^#^*P* < 0.05, ^##^*P* < 0.01 vs. BSA group; ^∗^*P* < 0.05, ^∗∗^*P* < 0.01 vs. AGEs group; *^Δ^P* < 0.05, *^ΔΔ^P* < 0.01 vs. AGEs + TSF group. Statistically analyzed via a one-way ANOVA with Dunnett's correction.

The downstream signaling pathway cascade of TRPC6 involves a Ca^2+^-dependent Calpain/Calcineurin signal transduction, which usually activates NFATCs. NFATC family members (NFATC1 through NFATC4) can be dephosphorylated by Calpain/Calcineurin signaling, resulting in their translocation to the nucleus to exert their function. The important transcriptional target of NFATCs is TRPC6 itself, which results in a harmful feedforward loop. In our study, we initially observed the expression of NFATC2 and 3 in AGEs-stimulated primary mice podocytes. Immunofluorescence morphology results revealed that AGEs-stimulated podocytes increased the percentage of NFATC2 translocated to the nucleus rather than NFATC3, and translocation could be reduced by TSF, while OAG attenuated its efficacy (Figures 8(a) and 8(b)). Therefore, we initially concluded that TSF reduced TRPC6-dependent Ca^2+^ accumulation, loss of talin1, and NFATC 2 nuclear translocation in primary mice podocytes stimulated by AGEs.

## 4. Discussion

In this study, a decreasing trend in the podocyte marker protein nephrin in *db/db* mice was observed; although, there was no significant difference in protein expression and localization compared with *db/m* control mice. The reduction in podocyte WT1 protein was more significant. Excluding some hereditary kidney diseases, foot process effacement is thought to help damaged podocytes attach to GBM by actin-cytoskeleton rearrangement, covering the exposed GBM due to partial podocyte exfoliation; it is an adaptive and reversible reaction to injury stimuli [46]. If the actin-cytoskeleton is repaired, early podocyte damage can be recovered to allow the foot process to branch again into their interdigitating pattern [2]. Thus, we hypothesized that partial foot process effacement of *db/db* mice in this study had not seriously affected the structure of the SD of podocytes and the related protein nephrin. The decrease in WT1-positive cells in glomeruli suggests a partial loss of podocytes, which allows us to focus on the cell-matrix adhesion structure.

The decrease of podocyte number is the strongest predictor of DKD progression. Podocyte apoptosis and detachment are the possible reasons for the loss of podocyte. Because the former is rarely observed in *in vivo* studies, podocyte loss can be attributed to detachment caused by podocyte adhesion failure [47, 48]. Podocytes adhere to the GBM by combining the actin cytoskeleton with the heterodimeric transmembrane integrin adhesion receptors. There are 18 kinds of *α* subunits and 8 kinds of *β* subunits, which can form 24 integrins in different combinations. Since the integrin cytoplasmic domain lacks actin-binding sites and catalytic activity, it is necessary to interact with cytoskeletal, adaptor and signaling molecules to form focal adhesion [49]. More than 200 components have been found at focal adhesions [50], including different receptor classes, linker molecules, GTPases, kinases, and phosphatases, collectively known as the adhesome. Focal adhesion provides a physical connection for podocytes to adhere to the GBM and regulates intracellular and extracellular signal transduction and actin network remodeling, thus controlling cell functions and morphologies [51]. Integrin activation is essential for the initiation of focal adhesions [52]. Talin is a large, 270 kDa protein with 18 domains and binds to at least 11 different focal adhesion components [53]. Compared with other focal adhesion proteins, talin has a special role as it binds to the cytoplasmic domain of the *β* integrin subunit, thereby activating the entire integrin that is capable of high-affinity interactions with extracellular matrix ligands [54]. At the same time, the active talin engaged with integrin can span the entire structure of focal adhesion, not only can mediate the signal transmission from the inside out but also to act as a mechanical sensor, which is essential for regulating the maturity and stability of focal adhesion [55, 56]. Studies have shown that the absence of *β*1 or *α*3 integrin in podocytes can lead to severe proteinuria [57, 58], and abnormal loss of talin1 affects the activation of *β*1 integrin, adhesion complex dynamics, and adhesion ability [8]. To ascertain the importance of proteins regulating focal adhesion in podocyte, we interrogated the Nephroseq v5 transcriptomic database. We observed that there was a striking negative correlation between the glomerular *TLN1* (talin1) expression with the body weight in *db/db* mice (Figure S4). Our results showed that TSF could significantly improve the body weight of *db/db* mice, while the irbesartan treatment did not exhibit the effect on the body weight of *db/db* mice. Furthermore, we observed that the glomerular *TLN1* expression negatively correlated with an increase of serum creatinine in the chronic kidney disease (CKD) cohort (Figure S4), our previous work demonstrated that TSF could significantly improve the serum creatinine level in the DKD patients [14]. These findings provided the impetus to further examine the importance of glomerular talin1 in the pathogenesis of DKD.

Talin1 has a strong positive correlation with the severity of proteinuria, reduction in cell adhesion, and podocyte actin-cytoskeletal disorganization [8]. To the best of our knowledge, our research is the first to explore the role of podocyte talin1 in DKD. We initially found that there was a significant loss in podocyte talin1 in *db/db* mice and AGEs-stimulated primary mice podocyte, which could be restored by TSF treatment. Meanwhile, increased cell migration and actin-cytoskeleton rearrangement caused by reduced talin1 were also seen in AGEs-induced primary mice podocytes. Talin1 in podocytes appears to be an important factor in the pathogenesis of DKD. As the key adhesion protein of the cell-matrix adhesion complexes, talins bind and activate integrins, coupling them to the actin cytoskeleton [59]. The two isoforms of talins in vertebrates are talin1 and talin2. Talin1 is closely linked to cell adhesion [60] and early embryonic development [61]. Talin1 function most likely predominates in normal podocyte physiology based on the evidence that podocyte-specific deletion of talin1 leads to severe podocyte injury and progressive proteinuria, while ablation of podocyte talin2 in mice lacking talin1 does not significantly change its phenotype [8].

AGEs are the nonenzymatic glycation of proteins caused by the long-term high glucose levels. AGEs have been demonstrated to be closely related to the development and progression of DKD, and can be involved in podocyte injury, endothelial cell damage, and renal fibrosis, and ultimately lead to impaired renal function [41, 42]. AGEs have been extensively used to explore the mechanism of podocyte injury in DKD. The enhancement of podocyte migration ability has been implicated as an injury phenotype. In renal pathology induced by adriamycin or unilateral ureteral ligation, dynamic podocyte movement can be observed [62]. Pan and colleagues [63] showed that SRGAP2a expression in podocytes of DKD patients and *db/db* mice was significantly reduced compared to normal controls. Overexpression of SRGAP2a could reverse previously abnormal actin cytoskeletal rearrangement, inhibit podocyte migration, and alleviate foot process effacement by inhibiting over-activated Rho A and Cdc42. In this study, we found that AGEs caused an abnormal increase in podocyte migration. We also observed increased formation of lamellipodia and filopodia, suggesting that rearrangement of the actin cytoskeleton in podocytes could be induced by AGEs. These findings are consistent with previously published reports [64–67]. The decreased expressions of talin1, integrin *β*1, and paxillin in primary mice podocytes after treatment with AGEs suggested that AGEs could cause the impairment in the cell-matrix adhesion ability of podocytes. TSF had a significant effect on the improvement of expressions of talin1, integrin *β*1, and paxillin in podocytes damaged by AGE stimulation. TSF also inhibited the migration phenotype of podocytes and alleviated the disorder of actin cytoskeleton. These effects of TSF are beneficial to the maintenance of the glomerular filtration barrier by improving the adhesion ability of podocytes.

Podocyte hypertrophy, foot process effacement, and the loss of podocytes in DKD are closely related to the abnormal expression of TRPC6 in podocytes, but little is known about its mechanism [68]. Cleavage of talin1 has been shown to be associated with TRPC6-dependent Ca^2+^ accumulation in podocyte injury [13]. Also, knockdown of *TRPC6* also led to decreased cleavage of talin1 [9]. This suggests that TRPC6-dependent Ca^2+^ accumulation is involved in the cleavage of talin1. Chen and colleagues [69] demonstrated that AGEs could induce an increase in [Ca^2+^]_i_ through influx of extracellular Ca^2+^ in podocytes. However, little is known about the relationship between AGEs and TRPC6 in this pathophysiologic process. In the current study, we found increased TRPC6 expression in both *db/db* mice and AGEs-stimulated primary mice podocytes. The intracellular Ca^2+^ level of podocytes regulated by TRPC6 plays a key role in maintaining the stability of the actin-cytoskeleton structure and podocyte injury [70]. Although the mechanism for TRPC6 activation remains unclear, abnormal changes in angiotensin II and reactive oxygen species (ROS) in the DKD setting can stimulate overexpression of TRPC6 in podocytes. This causes a large amount of Ca^2+^ influx into podocytes, resulting in foot process effacement, podocyte loss, and other damage, which eventually leads to proteinuria development. Our results showed that there was a significant increase in Ca^2+^ level in AGEs-treated podocytes compared with the control, and this increase was attenuated by TSF and the TRPC6 inhibitor SAR7334. This indicated that increased intracellular Ca^2+^ accumulation induced by TRPC6 is involved in podocyte injury caused by AGEs, and that TSF could reduce TRPC6-mediated Ca^2+^ accumulation.

Decrease in talin1 protein, increase in cell migration, and disorganization of F-actin in podocytes were also observed with the activation of TRPC6. The F-actin pattern determined by phalloidin staining transformed from more than 90% of cell area filled with thick cables or at least two thick cables running under the nucleus into no thick cables or no cables visible in the central area of the cell after the podocytes were stimulated by AGEs. These pattern changes are similar to the results reported in other studies, that TRPC6 KO podocytes display less motility, more adhesiveness and more actin stress fibers. These changes are alleviated after the reexpression of wtTRPC6 [9]. However, previous studies also showed the role of TRPC6 in regulating Rho A activation, stress fiber contraction, and motility [71, 72]. This difference may be attributed to the difference in *TRPC6* knockout level caused by siRNA technology used in previous studies, where some preservation of expression is seen. Our *in vitro* cell culture results showed that TSF could inhibit the increase in TRPC6 expression, decrease in talin1, and disorganization of actin-cytoskeleton. This effect could also be attenuated by the TRPC6 agonist OAG. These results suggest that TSF protects talin1 by inhibiting TRPC6 activation. Although much progress has been made in the development of TRPC6 agonists, either OAG, GSK1702934A [73], piperazine derivatives [74], or pyrazolopyrimidines [75], subtype selectivity is still lacking within the TRPC3/6/7 group of ion channels. Thus, further studies and more convincing evidence are needed to elucidate the protective effect of TSF on talin1 by inhibiting TRPC6 activation in podocytes.

Additionally, we observed an increase in NFATC2 entry into nuclei in AGEs-stimulated primary mice podocytes, but not NFATC3. The NFATC family (including NFATC1 through NFATC4) is usually located in the cytoplasm as a potential form of hyperphosphorylation [76] and is mainly regulated by serine/threonine phosphatase calcineurin. Upon activation, NFATCs can undergo dephosphorylation into the nucleus to perform their functions. The important transcriptional target of NFATCs is TRPC6 itself, which results in a harmful feedforward loop [77]. NFATCs are expressed in a variety of cell types, including podocytes. It has been shown that NFATC2 podocyte-specific overexpression leads to proteinuria and glomerular sclerosis [78]. Further studies [79, 80] have also found a significant increase in NFATC2 activation in podocytes of *db/db* mice and could be inhibited by the NFAT inhibitor peptide (11R-VIVIT). High glucose-induced mice podocytes also activate NFATC2 in a time- and dose-dependent manner and is prevented by *Nfatc2* gene knockdown. In our study, AGEs-stimulated primary mice podocytes increased intracellular Ca^2+^ concentration, leading to subsequent nuclear accumulation of NFATC2. We only explored the occurrences in NFATC2 and 3, as abundant literature on these two proteins provided the foundation for our investigation. Given our findings, we believe that TRPC6-dependent Ca^2+^ accumulation is involved in DKD podocyte injury, and the protective effect of TSF on podocytes may be through inhibition of TRPC6 activation and protection of talin1 from loss. However, TSF is a complex Chinese Medicine compound with multiple active ingredients. It is indeed difficult to accurately explain the protective effect of TSF on DKD with one mechanism. Therefore, it is important to explore all the possible biochemical interactions of TSF in podocytes and to clarify how the compounds interact with podocytes. This will be the focus of our further research.

## 5. Conclusion

In conclusion, the present study demonstrated that TSF could alleviate podocyte injury and reduce proteinuria induced by DKD, the mechanism of which might be mediated by the regulation of TRPC6/Talin1 pathway in podocytes, following an improvement in the podocyte-matrix adherence. These findings may be beneficial to our understanding of the role of TSF in protecting podocytes and further enrich our knowledge of the mechanism of podocyte injury in DKD.

## Data Availability

The data used to support the findings of this study are available from the corresponding authors upon request.

## Supplementary Materials

Supplementary Materials Table S1: compounds detected in TSF. Table S2: compounds detected in the serum of TSF treated mice. Figure S1: immunoblotting of TRPC6 and talin1 in MPC5 cells treated with scramble siRNA or *Trpc6* siRNA. Figure S2: immunohistochemical staining and quantitative analysis of collagen type I. Figure S3: the effect of irbesartan in combination with AGEs in wound healing assay. Figure S4: glomerular *TLN1* expression in *db/db* mice and CKD patients.
