# Supplementary material for: Protective Role of Tangshen Formula on the Progression of Renal Damage in db/db Mice by TRPC6/Talin1 Pathway in Podocytes
Source: J Diabetes Res. 2020 Sep 16;2020:3634974. doi: 10.1155/2020/3634974 (PMC7519445; doi:10.1155/2020/3634974)
Supplement: Supplementary Materials — Table S1: compounds detected in TSF. Table S2: compounds detected in the serum of TSF treated mice. Figure S1: immunoblotting of TRPC6 and talin1 in MPC5 cells treated with scramble siRNA or Trpc6 siRNA. Figure S2: immunohistochemical staining and quantitative analysis of collagen type I. Figure S3: the effect of irbesartan in combination with AGEs in wound healing assay. Figure S4: glomerular TLN1 expression in db/db mice and CKD patients. [file 3634974.f1.docx]

**Table 1 Compounds detected in TSF**

|  | Name | | MW | Formula | CAS |  |
| --- | --- | --- | --- | --- | --- | --- |
| 1 | 7-O-Methyl morroniside | 420.411 | | C_18_H_28_O_11_ | 41679-97-4 | |
| 2 | 7-O-Ethylmorroniside | 434.438 | | C_19_H_30_O_11_ | 945721-10-8 | |
| 3 | 7-Ketologanin | 388.369 | | C_17_H_24_O_10_ | 152-91-0 | |
| 4 | (-)-Epicatechingallate | 442.376 | | C_22_H_18_O_10_ | 1257-08-5 | |
| 5 | Ginsenoside Rg1 | 801.024 | | C_42_H_72_O_14_ | 22427-39-0 | |
| 6 | Hesperidin | 610.565 | | C_28_H_34_O_15_ | 520-26-3 | |
| 7 | Nobiletin | 402.399 | | C_21_H_22_O_8_ | 478-01-3 | |
| 8 | Emodin | 270.24 | | C_15_H_10_O_5_ | 518-82-1 | |
| 9 | Emodin 8-glucoside | 432.381 | | C_21_H_20_O_10_ | 23313-21-5 | |
| 10 | Rhein | 284.223 | | C_15_H_8_O_6_ | 478-43-3 | |
| 11 | Monomelittoside | 362.33 | | C_15_H_22_O_10_ | 20633-72-1 | |
| 12 | Rehmannioside D | 686.613 | | C_27_H_42_O_20_ | 81720-08-3 | |
| 13 | (+)-Taxifolin | 304.254 | | C_15_H_12_O_7_ | 480-18-2 | |
| 14 | Sennoside C | 848.763 | | C_42_H_40_O_19_ | 37271-16-2 | |
| 15 | Quercetin | 302.238 | | C_15_H_10_O_7_ | 117-39-5 | |
| 16 | Astragaloside a | 784.983 | | C_41_H_68_O_14_ | 83207-58-3 | |
| 17 | Hyperoside | 464.379 | | C_21_H_20_O_12_ | 482-36-0 | |
| 18 | Geniposide | 388.369 | | C_17_H_24_O_10_ | 24512-63-8 | |
| 19 | Tangeretin | 372.373 | | C_20_H_20_O_7_ | 481-53-8 | |
| 20 | Aloe-emodin | 270.24 | | C_15_H_10_O_5_ | 481-72-1 | |
| 21 | Loganin | 390.385 | | C_17_H_26_O_10_ | 18524-94-2 | |
| 22 | Formononetin glucoside | 430.4 | | C_22_H_22_O_9_ | 486-62-4 | |
| 23 | Formononetin | 268.26 | | C_16_H_12_O_4_ | 485-72-3 | |
| 24 | Overbascoside | 624.592 | | C_29_H_36_O_15_ | 61276-17-3 | |
| 25 | Calycosin | 284.3 | | C_16_H_12_O_5_ | 20633-67-9 | |
| 26 | Calycosin-7-O-beta-D-glucopyranoside | 446.4 | | C_22_H_22_O_10_ | 20633-67-4 | |
| 27 | Gallic acid | 170.12 | | C_7_H_6_O_5_ | 149-91-7 | |
| 28 | Morroniside | 406.384 | | C_17_H_26_O_11_ | 25406-64-8 | |
| 29 | Adehydrodicatechin A | 578 | | C_30_H_26_O_12_ | -- | |
| 30 | Kaempferol 3-sophoroside 7-rhamnoside | 756.663 | | C_33_H_40_O_20_ | 93098-79-4 | |
| 31 | Eriodictioside | 596.533 | | C_27_H_32_O_15_ | 13463-28-0 | |
| 32 | Neohesperidin | 610.565 | | C_28_H_34_O_15_ | 13241-33-3 | |
| 33 | Neoeriocitrin | 596.538 | | C_27_H_32_O_15_ | 13241-32-2 | |
| 34 | Isoverbascoside | 624.592 | | C_29_H_36_O_15_ | 61303-13-7 | |
| 35 | Ajugol | 348.348 | | C_15_H_24_O_9_ | 52949-83-4 | |
| 36 | Naringin | 580.539 | | C_27_H_32_O_14_ | 10236-47-2 | |
| 37 | Naringenin | 272.256 | | C_15_H_12_O_5_ | 480-41-1 | |
| 38 | Narirutin | 580.539 | | C_27_H_32_O_14_ | 14259-46-2 | |
| 39 | Sweroside | 358.343 | | C_16_H_22_O_9_ | 14215-86-2 | |
| 40 | Catalpol | 362.331 | | C_15_H_22_O_10_ | 2415-24-9 | |

Note: -- means no CAS number.

Table 2 Compounds detected in the serum of TSF treated mice

|  | Name | Structure | MW | Formula | CAS |
| --- | --- | --- | --- | --- | --- |
| 1 | Hesperiden | 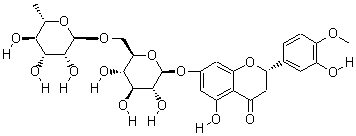 | 610.5606 | C_28_H_34_O_15_ | 520-26-3 |
| 2 | Astragaloside IV | 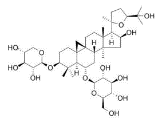 | 784.9825 | C_41_H_68_O_14_ | 83207-58-3 |
| 3 | Formononetin glucoside | 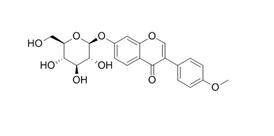 | 430.4047 | C_22_H_22_O_9_ | 486-62-4 |
| 4 | Formononetin | 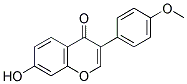 | 268.2641 | C_16_H_12_O_4_ | 485-72-3 |
| 5 | Calycosin | 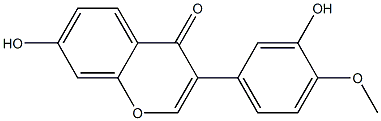 | 284.6235 | C_16_H_12_O_5_ | 20575-57-9 |
| 6 | Calycosin-7-O-beta-D-glucopyranoside | 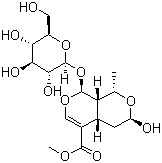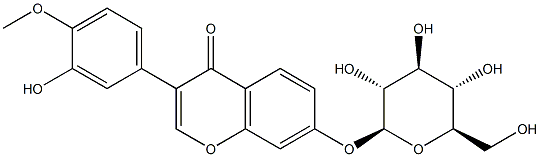 | 446.4041 | C_22_H_22_O_10_ | 20633-67-4 |
| 7 | Morroniside |  | 406.3817 | C_17_H_26_O_11_ | 25406-64-8 |
| 8 | Ginsenoside RG1 | 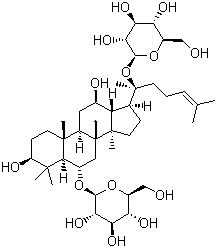 | 801.0127 | C_42_H_72_O_14_ | 22427-39-0 |
| 9 | Neohesperidin | 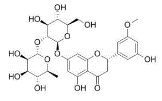 | 610.5606 | C_28_H_34_O_15_ | 13241-33-3 |
| 10 | Ajugol | 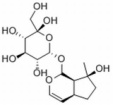 | 348.3457 | C_15_H_24_O_9_ | 52949-83-4 |
| 11 | Naringin | 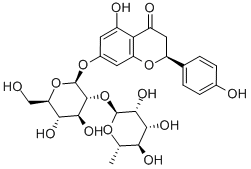 | 580.5346 | C_27_H_32_O_14_ | 10236-47-2 |
| 12 | Narirutin | 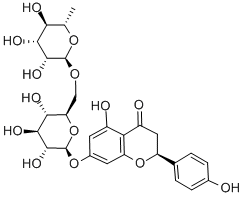 | 580.5346 | C_27_H_32_O_14_ | 14259-46-2 |

**Figure S1** **Immunoblotting of TRPC6 and talin1 in MPC5 cells treated with scramble siRNA (control ) or *Trpc6* siRNA**


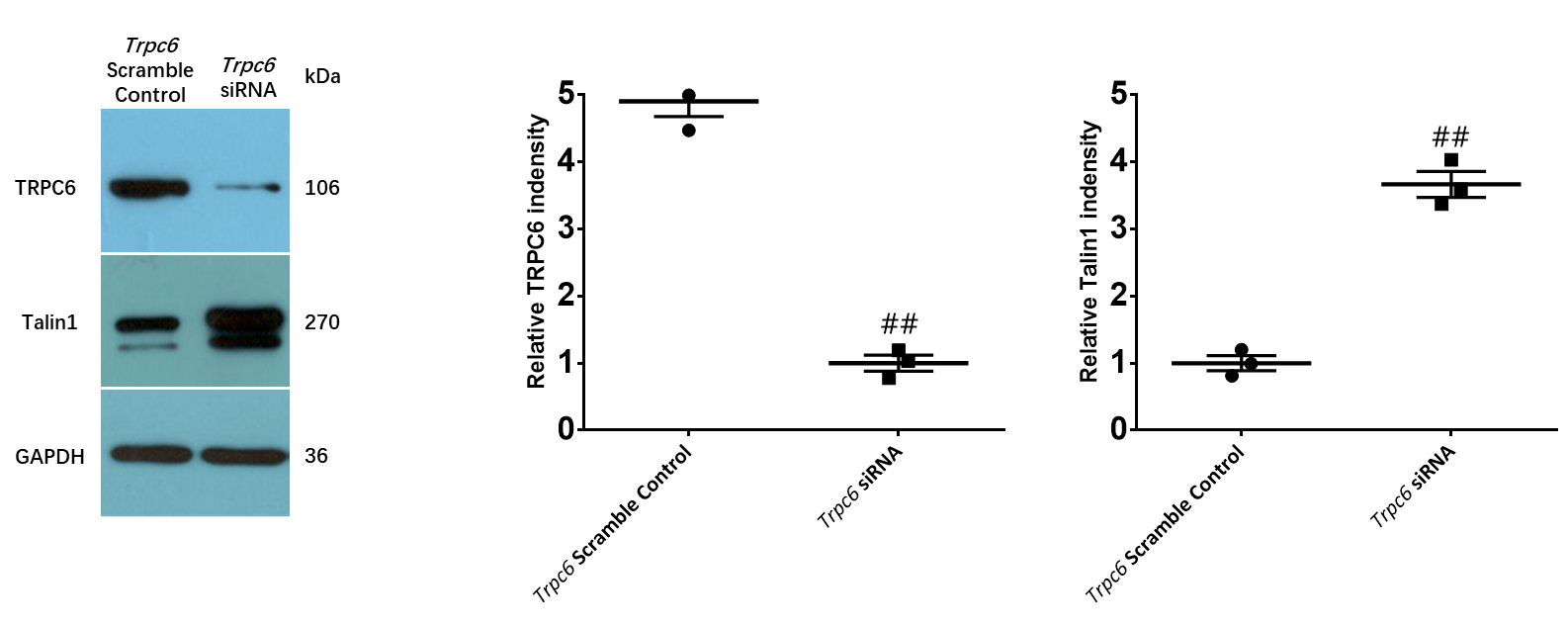


siRNA against *Trpc6* was purchased from Generay (Shanghai Generay Biotech Co., Ltd) (*n*=3 experiments). The data were expressed as the mean ± SEM. ^##^*P*＜0.01 vs. *Trpc6* scramble control group. Statistically analyzed via a two-tailed *t* test.

*Trpc6* Forward: 5'-GGAGGCAAUCCUCAACCAUTT-3'

Reverse: 5'-AUGGUUGAGGAUUGCCUCCTT-3'

**Figure S2 Immunohistochemical staining and quantitative analysis of collagen type I**


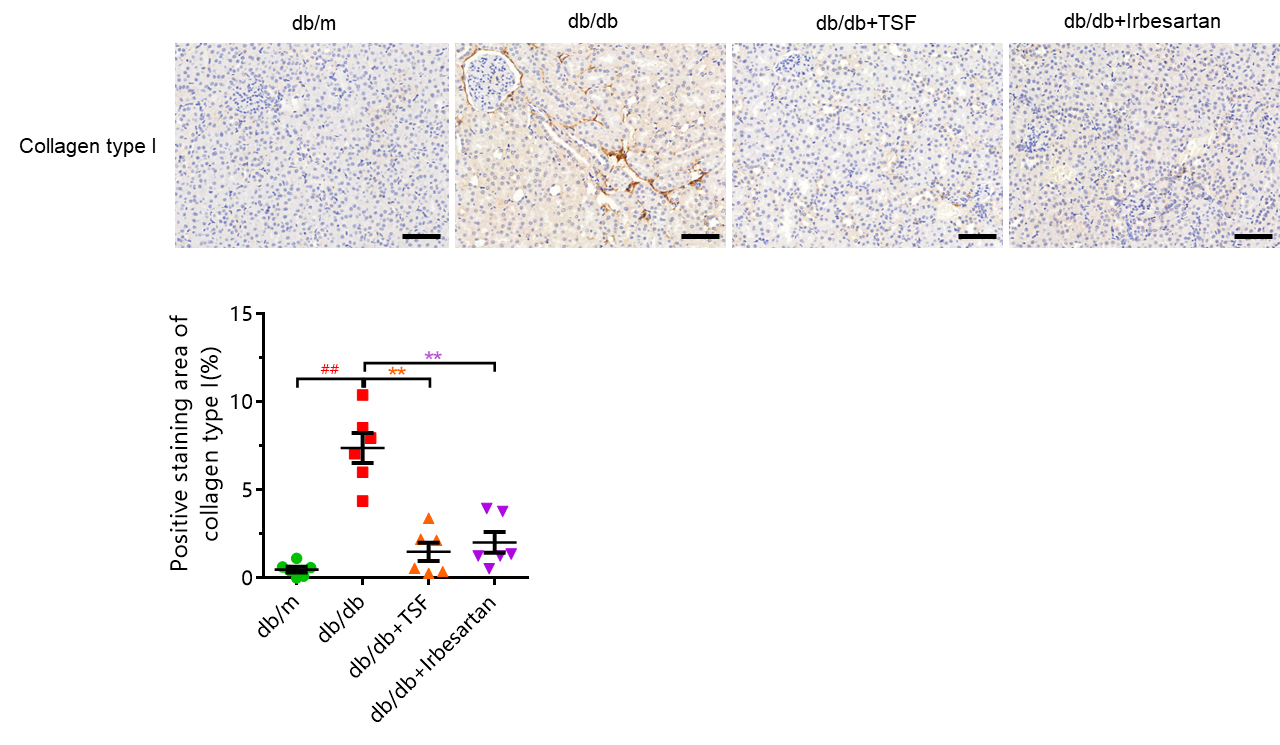


Representative immunohistochemical staining and quantitative analysis of collagen type I (scale bars, 100 μm) (20 glomeruli were randomly evaluated per mouse, *n*=6). The data were expressed as the mean ± SEM. ^#^*P*＜0.05, ^##^*P*＜0.01 vs. *db/m* group; **P*＜0.05, ***P*＜0.01 vs. *db/db* group. Statistically analyzed via a one-way ANOVA with Dunnett’s correction.

**Figure S3 The effect of irbesartan in combination with AGEs in wound healing assay**


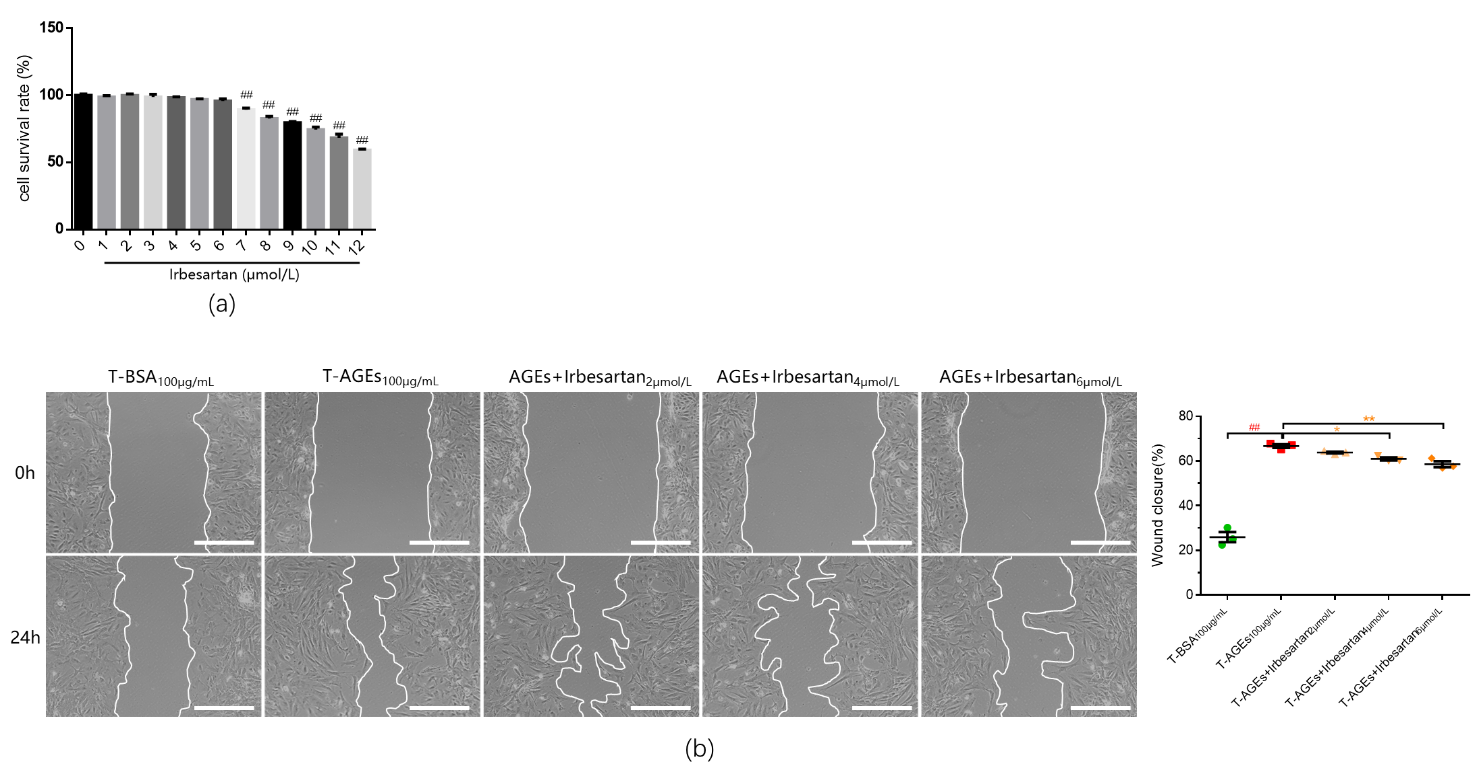


(a) The viability of primary mice podocytes was determined with MTT assay (*n*=3 experiments). (b) Wound healing assay and quantitative analysis using different doses of irbesartan for 24 hours (scale bars, 100 μm) (*n*=3 experiments). The data were expressed as the mean ± SEM of three independent experiments performed in triplicate. ^#^*P*＜0.05, ^##^*P*＜0.01 vs. BSA group; **P*＜0.05, ***P*＜0.01 vs. AGEs group. Statistically analyzed via a one-way ANOVA with Dunnett’s correction.

**Figure S4 Reduced glomerular *TLN1* expression is observed with an increase of body weight in the *db/db* DKD mouse model; reduced glomerular *TLN1* expression with an increased serum creatinine level is observed in the CKD patients.**

To ascertain the importance of proteins regulating focal adhesion in podocyte, we interrogated the Nephroseq v5 transcriptomic database. We observed that there was a striking negative correlation between the glomerular *TLN1* (talin1) expression with the body weight in *db/db* mice (DKD model), also glomerular *TLN1* expression negatively correlated with an increase of serum creatinine in the chronic kidney disease (CKD) cohort.
